# Optimization of SiC–TiC Composite Manufacturing by Electroconsolidation Method

**DOI:** 10.3390/ma18092062

**Published:** 2025-04-30

**Authors:** Vyacheslav Ivzhenko, Jolanta Natalia Latosińska, Edvin Hevorkian, Miroslaw Rucki, Tamara Kosenchuk, Natalia Shamsutdinova, Tadeusz Szumiata, Volodymyr Chishkala, Arturas Kilikevicius

**Affiliations:** 1 V.M. Bakul Institute of Superhard Materials, National Academy of Sciences of Ukraine, Avtozavodska Str. 2, 04074 Kyiv, Ukraine; ivv@ism.kiev.ua (V.I.); avl@ism.kiev.ua (T.K.); 2 Faculty of Physics and Astronomy, Adam Mickiewicz University, Uniwersytetu Poznańskiego 2, 61-614 Poznań, Poland; 3 Faculty of Mechanical Engineering, Casimir Pulaski Radom University, Stasieckiego 54-B1, 26-600 Radom, Poland; e.gevorkian@urad.edu.pl (E.H.); t.szumiata@urad.edu.pl (T.S.); 4 Institute of Mechanical Science, Vilnius Gediminas Technical University, Sauletekio al. 11, LT-10223 Vilnius, Lithuania; m.rucki@urad.edu.pl (M.R.); arturas.kilikevicius@vilniustech.lt (A.K.); 5 Plasma Tech LLC, Chmelnitske Sh. 13, 21036 Vinnytsia, Ukraine; shamsut.natalka49@gmail.com; 6 Department of Reactor Engineering Materials and Physical Technologies, V.N. Karazin Kharkiv National University, m. Nezalezhnisti 4, 61022 Kharkiv, Ukraine; vchishkala@ukr.net

**Keywords:** silicon carbide, titanium carbide, electric spark sintering, temperature, pressure, structure, porosity, manufacturing optimization

## Abstract

Modern SiC-based materials are of paramount importance in that they serve as wear-resistant and thermal protectors and as next-generation single-photon sources for photonic and quantum solutions. Efforts are underway to identify more efficient methods of manufacturing SiC-based ceramic materials. The objective of this paper is to provide a description of the optimization of sintering SiC–TiC composites by the electroconsolidation method. The influence of titanium carbide content on the physical and mechanical properties of SiC–TiC composites obtained by spark plasma sintering (SPS) at a pressure of 45 MPa was studied. It was found that compared to sintered silicon carbide, the porosity of composites with 40 mol% TiC decreased from ~30% to 0%, the crack resistance increased from 2.9 to 6.1 MPa × m^0.5^, and the hardness increased from 2.9 to 21.5 GPa. The influence of sintering temperature and holding time on SiC–TiC composites’ physical and mechanical properties during sintering at a pressure of 45 MP was also investigated. An increase in temperature from 1900 °C to 2000 °C resulted in an approximately 30% rise in the composite hardness. An extension of the time allotted for the sintering process from 30 to 45 min resulted in a decrease in both the fracture toughness and hardness of the material. The utilization of two- and three-dimensional vector spaces of material features was proposed as a novel methodology for the description of manufacturing process optimization.

## 1. Introduction

Functional materials employed in modern sensing devices exhibit a wide range of physical properties, including piezoelectricity, pyroelectricity, and sensitivity to external stimuli such as temperature, pressure, and chemical environments. Materials such as barium titanate, zinc oxide, and various compositions based on ferroelectric and piezoelectric compounds have drawn significant research attention due to their high sensitivity and operational stability under diverse conditions [1,2,3]. Their performance—including dielectric response, thermal conductivity, and structural stability—plays a crucial role in defining sensor output characteristics, sensitivity, and accuracy [4,5]. Furthermore, the surface morphology and nanostructural features of these materials greatly influence their interaction with the surrounding environment. These findings have significant implications for the development of next-generation sensors, which show considerable potential in terms of enhanced selectivity and miniaturization potential [6].

In recent years, silicon carbide (SiC, carborundum) has emerged as a material of choice for a wide range of contemporary applications due to the unique properties it exhibits [7]. It has been utilized as a fundamental component in the fabrication of bulk materials and coatings. SiC has become a prevalent material in the manufacturing of power electronics, electric vehicles, and solar and wind turbine systems [8], and its primary function is enhancing the efficiency of components. Its heat resistance, high resistance to radiation, and low neutron absorption rate render it an optimal solution as a thermal protector, especially for the construction of nuclear reactors. SiC-based photodetectors have been shown to exhibit large gains in, as well as high sensitivity and a rapid and consistent response to, α, β, neutron, X/γ, and UV radiation [9,10]. Furthermore, in the domain of abrasive, grinding, and cutting tools, SiC has been demonstrated to offer enhanced chemical and mechanical durability. Recently, SiC was tested for its suitability in the production of prototypes of implants and prostheses [11,12], which, in addition to durability, must demonstrate both biocompatibility and corrosion resistance. Recent interest in SiC has seen a marked escalation following the discovery of point defects in its crystal lattice (the so-called color centers), which can serve as next-generation single-photon sources [13,14,15]. Single-photon sources are a key element in the practical implementation of photonic and quantum computing solutions. In this extensive range of applications, the efficient manufacturing of novel materials based on SiC is of particular importance.

Silicon carbide appears in more than 250 polymorphic forms, including various amorphous phases (thin films and fibers) as well as crystalline polytypes (differing in so-called A, B, and C stacking) [16]. There are two crystalline polymorphs, namely, the high-temperature stable α-form (hexagonal/rhombohedral; 2H-SiC, 4H-SiC, 6H-SiC, nH-SiC, 15R, and 21R), which is most common, and the low-temperature stable β-form (cubic; 3CSiC) [17]. The structural distinctions between both forms can be attributed to the primitive unit cell type, the order of alternation and periodicity of layers along one crystallographic axis, and the degree of hexagonality.

The main advantage of SiC is its high hardness, surpassed only by diamond, cubic boron nitride, and boron carbide [18], but its low fracture toughness of 3–4 MPa⋅m^0.5^ makes it less reliable in critical applications. However, SiC exhibits a low self-diffusion coefficient due to the dominating C—Si covalent bond, which affects its densification process and comprises 88% of its bonds, with ionic bonds comprising the remaining 12%. Thus, it is necessary to select an appropriate sintering method and thermodynamic simulation by adding certain elements that do not promote the decomposition of SiC at elevated temperatures [19]. In particular, alumina, yttria, and zirconia may serve as additives for sintered silicon carbide-based composites with high mechanical properties [20,21].

Titanium carbide (TiC) is a metallic mineral known to become plastic at elevated temperatures. TiC fcc crystals have 12 slip systems and can undergo slip deformation at high temperatures. TiC exhibits excellent properties, including a high melting point (3140 °C) and hardness (HV = 30.3 GPa) [22], which collectively serve to secure its classification as a covalently bonded ceramic. Nevertheless, the low level of toughness exhibited by this material results in its resemblance to other non-oxide ceramics. Elevated temperatures have been shown to cause a substantial increase in the size of the grains, which has a deleterious effect on the fracturing and bending strength of this material. The inferior mechanical property of TiC, i.e., its intrinsic brittleness, exerts a considerable restriction on its utilization. However, the use of TiC in the production of wear-resistant tools as a conductivity-improving additive and nucleating agent has been well documented. In recent years, considerable research activity has been dedicated to the synthesis of titanium carbide (TiC)-based nanoceramics.

It has long been established that the addition of certain amounts of TiC powder of varying fineness has a positive effect on the mechanical and oxidation properties of α-SiC [23]. A high-toughness SiC–TiC particulate composite material was produced through a combination of SiC and TiC, both of which are recognized as having low levels of toughness when utilized individually. Recent findings have indicated that the addition of titanium carbides improves wear resistance by ca. 25% compared to that of 90 wt.% SiC–10 wt.% B_4_C [24]. Research on high-density SiC–(Ti_x_Zr_1−x_)B_2_ and SiC–TiB_2_ composite ceramics fabricated by solid-state spark plasma sintering demonstrated that materials of relative density 99.97% exhibited higher flexure strength and fracture toughness by 242.6% and 53.6%, respectively, than single-phase SiC ceramics [25]. The authors of the abovementioned study concluded that the toughening effect could be attributed to the shell–core structure.

Even though additive manufacturing (AM) techniques for the fabrication of silicon carbide ceramics are attracting increasing interest [26], the sintering methods of various modifications remain dominant in this area [27,28]. The parameters of the sintering process enable control over the densification process and hence the resulting structure and related mechanical properties of SiC-based composites.

In previous research, the kinetics of the densification process was investigated in relation to SiC–TiC and SiC–VC systems [29]. It was found that the interface area between phases plays an important role in the densification process. Liu et al. [30] used molecular dynamic simulations to investigate the interaction of submicron particles of SiC during the sintering process. Particular attention was paid to the evolution of surface morphology and microstructure, radial distribution function, atomic displacement distribution, mean square displacement, and structural transformation, as well as the diffusion coefficient. The authors concluded that when the sintering time was increased, the dominant mechanism shifted from particle surface diffusion to interface diffusion, increasing atomic migration and interface energy consumption. Lei and co-authors [31] used the finite element method to predict and control the sintering processes of porous SiC cylindrical samples—particularly, temperature distribution, but also electrical and thermal fields. They found that the temperature increase in the core of the samples was proportional to their porosity, increasing from 1960 °C up to 2010 °C when porosity increased from 1% up to 60%.

Conventionally, composites are fabricated using spark plasma sintering (SPS) or hot-pressing (HP) methodologies. SPS has been shown to result in accelerated densification and, in many cases, reduced grain growth in comparison with conventional HP methods. Electroconsolidation, a process that employs an electric current to consolidate powder into a dense material, offers several significant advantages over both aforementioned densification methods. The advantages of this approach are numerous, including enhanced densification quality (obviating the necessity for coating), the capacity to produce fine-grained structures, the augmented mechanical properties of the ceramic (e.g., density, hardness, and fracture toughness), and energy efficiency in comparison to classical methods.

The present study is aimed at investigating the influence of different parameters of the sintering process on the mechanical properties of SiC–TiC ceramics fabricated using electroconsolidation. In particular, the effect of temperature and holding time on hardness and fracture toughness was investigated in relation to the titanium carbide percentage in the composites. The utilization of two- and three-dimensional vector spaces of material features was proposed as a novel methodology for the description of manufacturing process optimization.

## 2. Materials and Methods

### 2.1. Experimental Section

**Figure 1:**
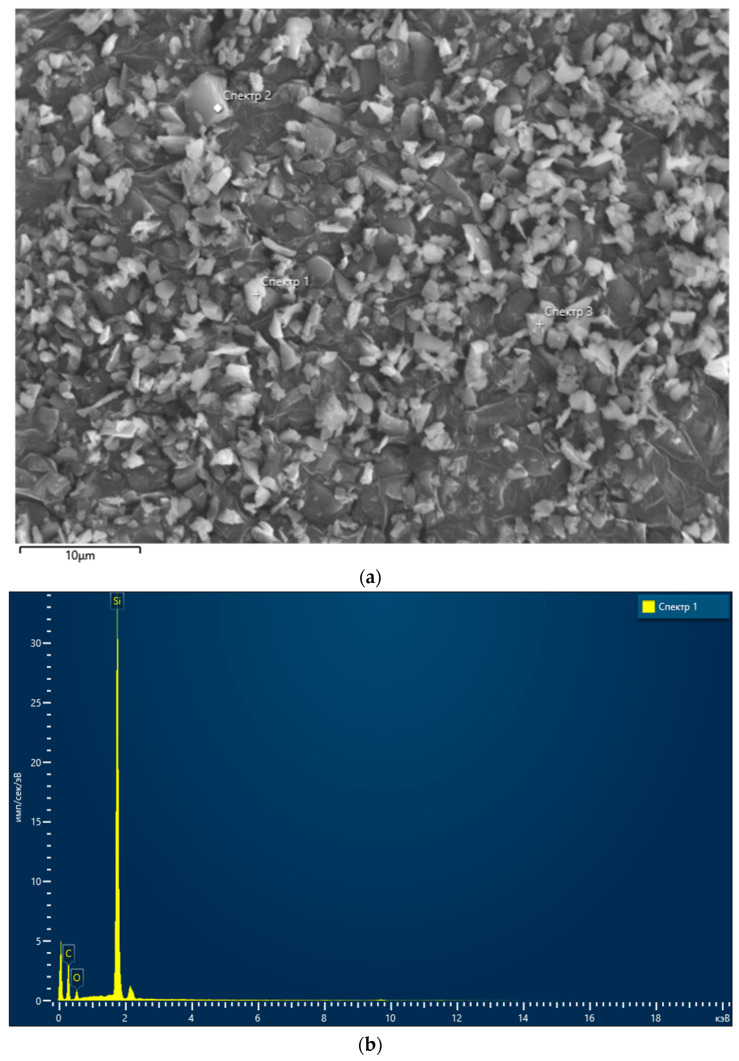
SEM-EDX analysis of the SiC powder: (**a**) SEM image; location of samples taken for the further analysis (Table 1) is marked as using numbers 1, 2, and 3; (**b**) composition.

In the course of the experiments, α–SiC powder grade M2 produced by Zaporizhzhia Abrasive Plant (Zaporizhzhia, Ukraine) was used. The powder, with an average particle size of 2 µm, contained ca. 98% of SiC and no more than 0.1 wt.% Fe, 1.5 wt.% O, and 0.4 wt.% C. Figure 1 presents an SEM image of the powder and an SEM–EDX (scanning electron microscope with energy-dispersive X-ray) analysis of the powders. Table 1 presents the composition of the analyzed SiC powder.

**Figure 2:**
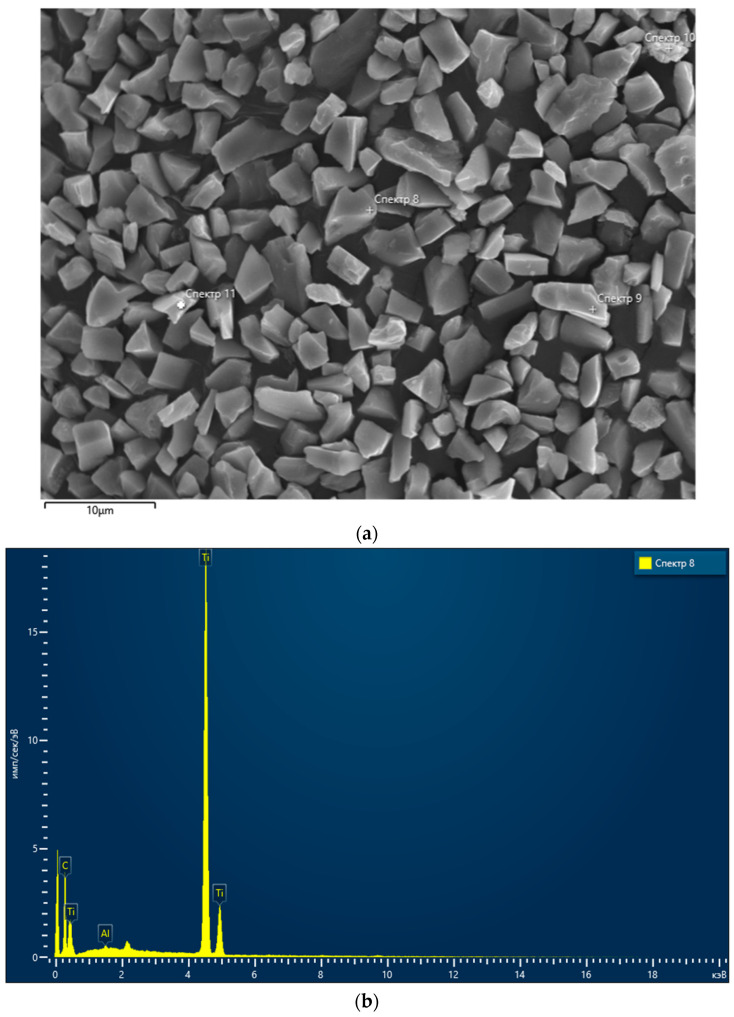
SEM-EDX analysis of the TiC powder: (**a**) SEM image; location of samples taken for the further analysis (Table 2) is marked as using numbers 11, 10, 9 and 8; (**b**) composition.

The additive was TiC powder with an average particle size of 4 µm. Its X-ray absorption spectroscopy (XAS) diffractogram is shown in Figure 2. Table 2 presents the respective composition of the TiC powder. 

TiC powder was added to SiC in proportions of 5, 20, 30, and 40% by mass. The two components were blended together for 24 h with a ball mill in a wet environment, using balls made of hot-pressed silicon carbide. After this, the samples were sintered in graphite molds using the electroconsolidation apparatus described in [32].

The high compaction of sintered silicon carbide requires high temperatures, high pressure, and a longer holding time than most refractory powders. In the case of the applied SPS apparatus, the strength of the graphite molds did not allow for an increase in the uniaxial pressure above 50 MPa. In addition, the powders were of a microscale and exhibited low sinterability compared to nanopowders, so that the holding time was prolonged to 5, 15, 30, and 45 min for two different sintering temperatures: 1900 °C and 2000 °C. For comparison, using submicron powders, it was possible to sinter the composition 80 wt.% SiC + 20 wt.% (Al_2_O_3_ + ZrO_2_) at a temperature of 1860 °C, under a uniaxial pressure of 30 MPa, with a holding time of 3 min [21]. The sintering process took place under a current of 5000 A and voltage 5 V, in a vacuum of 1.5 Pa, ensuring a heating rate of 300 °C/min. A uniaxial mechanical pressure of 45 MPa was applied after the temperature reached 1000 °C, and it was released 3 min after turning off the current. Specimens obtained in this process were of a cylindrical shape with a diameter ∅ 11 mm and a height of 5 mm.

Density and related porosity were determined according to the standard EN ISO 3369:2014. The Vickers hardness HV was measured under the load 150 N using the Matsuzawa MXT70 tester (Matsuzawa Co. Ltd., Akita, Japan). Indentation was examined with the NU–2E microscope produced by Carl Zeiss (Jena, Germany) with 750× zoom. From the indentation and the length of radial cracks, fracture toughness K_1c_ was calculated according to the well-known Evans and Charles equation [33].

The sintered samples were examined with the Tescan Vega 3 SBH EP (TESCAN, Brno, Czech Republic) scanning electron microscope (SEM). During measurement, the accelerating voltage was 20–30 kV in backscattered electron (BSE) and secondary electron (SE) modes at different magnifications. The resolution of this device is as high as 3.5 nm. The chemical composition of the samples was determined by element mapping, point analysis, and the scanning of a surface of 0.09–0.25 mm^2^. The application of the Bruker Quantax 610 M energy-dispersive X-ray spectroscope (EDS) (Bruker, Billerica, MA, USA) permitted the identification and quantification of elements in the range from B (z = 5) to Am (z = 95).

### 2.2. Material Feature Vector Approach

Conventionally, the evaluation of material quality is undertaken via manual analysis of a given set of properties, with these properties being determined experimentally. A more computationally oriented approach, which is an alternative to the aforementioned method, involves the use of dedicated n-dimensional feature vectors (vectors of features) in an n-dimensional feature vector space. The n-dimensional feature vectors constitute a distinct category of vectors, designated as such due to their capacity to articulate a set of features through a single mathematical quantity. The n-dimensional material feature vector can be defined as a numerical representation of material features, which is based on an ordered list of numerical values that characterize the given material. Thus, the material feature vector is constructed based on a tuple describing the set of properties for a given type of material. From a mathematical perspective, a vector is preferable to a tuple for the purposes of subsequent analysis. From a physical perspective, the material features are described in a uniform manner using material feature vectors combining grain size, hardness, and fracture toughness, i.e., the parameters which constitute the basis for the assessment of material quality. The angles and magnitudes describing these vectors are thus related directly to the material’s properties.

In classical data analysis, n-dimensional vectors that possess both direction and magnitude are employed as a convenient tool for numerical representation, thereby enabling a wide range of analyses. A plethora of methodologies exists for the comparison of vectors based on diverse mathematical metrics, including cosine or Euclidean ones.

The cosine similarity (CS) between the material feature vectors can be defined using the cosine metric as follows:

(1) $$d_{c}\left(p,q\right)=\frac{\sum_{i}p_{i}q_{i}}{{\left(\sum_{i}p_{i}^{2}\sum_{i}q_{i}^{2}\right)}^{1/2}}$$

where <p> = {p_i_} and <q> = {q_i_} are the material feature vectors. The numerical value of d_c_(p, q) ranges from 0 to 1, and d_c_(p, q) = 1 indicates the identity of vectors. The error value of d_c_(p, q) can be derived using the absolute value of the total differential:

(2) $$\left|\Delta d_{c}\left(p,q\right)\right|=\underset{i}{\sum }\frac{q_{i}\sum_{j\neq i}p_{j}^{2}-p_{i}\sum_{j\neq i}p_{j}q_{j}}{{\left(\sum_{i}p_{i}^{2}\right)}^{3/2}{\left(\sum_{i}q_{i}^{2}\right)}^{1/2}}\left|\Delta p_{i}\right|+\underset{i}{\sum }\frac{p_{i}\sum_{j\neq i}q_{j}^{2}-q_{i}\sum_{j\neq i}p_{j}q_{j}}{{\left(\sum_{i}p_{i}^{2}\right)}^{3/2}{\left(\sum_{i}q_{i}^{2}\right)}^{1/2}}\left|\Delta q_{i}\right|$$

where <Δp> = {Δp_i_} and <Δq> = {Δq_i_} are the error vectors.

The cosine metric is a useful tool for the purpose of comparing vectors based on their directional similarity. However, since the vectors are normalized, any differences in magnitude between them are rendered negligible.

The Euclidean distance (ED) between the material feature vectors can be defined using the Euclidean metric, also known as the L2 norm, as follows:

(3) $$d_{E}\left(p,q\right)={\left(\sum_{i}{\left(p_{i}-q_{i}\right)}^{2}\right)}^{1/2}$$

where <p> = {p_i_} and <q> = {q_i_} are the material feature vectors. The numerical value of d_E_(p, q) ranges from 0 to infinity, and d_E_(p, q) = 0 indicates the identity of vectors. The error value of d_E_(p, q) can be derived using the absolute value of the total differential:

(4) $$\left|\Delta d_{E}\left(p,q\right)\right|=2|\sum_{i}\left(p_{i}-q_{i}\right)|\left(\left|\Delta p_{i}\right|+\left|\Delta q_{i}\right|\right)$$

where <Δp> = {Δp_i_} and <Δq>{Δq_i_} are the error vectors.

The Euclidean metric facilitates the identification of more intricate relationships and consequently enables a more precise delineation of the similarity between vectors and the materials’ features. Unlike the cosine metric, it measures the absolute difference in vector values.

There is no universal rule for choosing between the Euclidean and cosine metrics, as their suitability largely depends on the nature of the data. From a mathematical standpoint, the utilization of hybrid approaches, incorporating both metrics, appears optimal. This approach is underpinned by the principle that each metric enables the evaluation of distinct aspects. Irrespective of the metric under consideration, the vector approach has the advantageous capability of capturing regularities, thus facilitating the optimization of material features. Nevertheless, cosine metrics, which do not capture deep relationships between materials, may be less accurate.

The application of a heat map technique provides a visual representation of features. It facilitates the capture of the most salient data by representing the magnitude of individual values within a dataset by color [34]. In this study, grid color-coded heat maps were used to facilitate the visualization of the similarity between material feature vectors.

The material feature approach described above was combined with use of mathematical metrics as similarity measures and heat maps for the visualization of the distance between vectors. It shares distant semantic and methodological connotations with the method used for the in-depth analysis and prediction of binding modes in protein–ligand complexes [34,35].

The validity of the material feature vector approach was verified through an additional analysis that utilized distinct sets of experimental data for a different material. The verification focused primarily on composites which have been the subject of recent research but constituted a set separate from SiC–TiC composites. For the abovementioned set of data, the classical analysis in conjunction with the results obtained based on material feature vectors yielded convergent results.

## 3. Results and Discussion

### 3.1. Structure of the Composite

**Figure 3:**
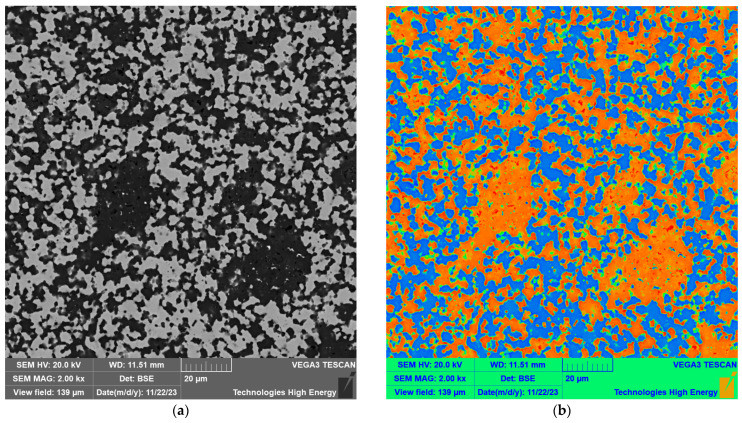

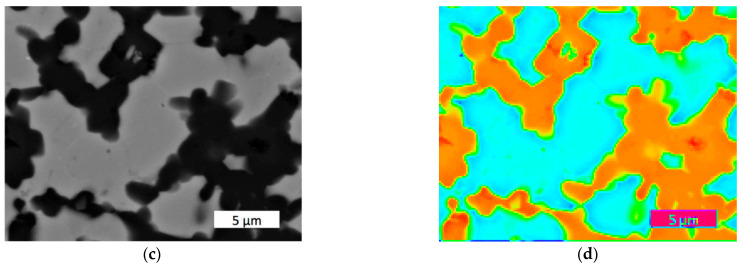
Microstructure of 60%SiC–40%TiC material sintered at 2000 °C under uniaxial pressure of 45 MPa for 30 min (**a**); the boundaries are better resolved using a heat map (SiC grains and TiC inclusions are depicted in orange and blue/cyan, respectively (**b**)). A section of each image has been cut out and enlarged to show the boundaries between grains (**c**,**d**). The color scale ranges from red to blue, with thresholds set at every 10% increment in image brightness.

The structure of the sintered material consisted of dark gray grains of the matrix SiC material and light gray inclusions of the titanium carbide. An example structure of the composition 60 wt.% SiC–40 wt.% TiC sintered at 2000 °C under a uniaxial pressure of 45 MPa for 30 min is shown in Figure 3a. A visual representation of the magnitude of individual values as colors is provided in Figure 3b. A section of each image has been cut out and enlarged to show the boundaries between grains in Figure 3c,d.

The heat map visualization shown in Figure 3d reveals boundaries between grains of SiC, depicted in orange, and inclusions of TiC, depicted in cyan. The scale bars are shown in the images. The same color scale was used for both figures.

**Figure 4:**
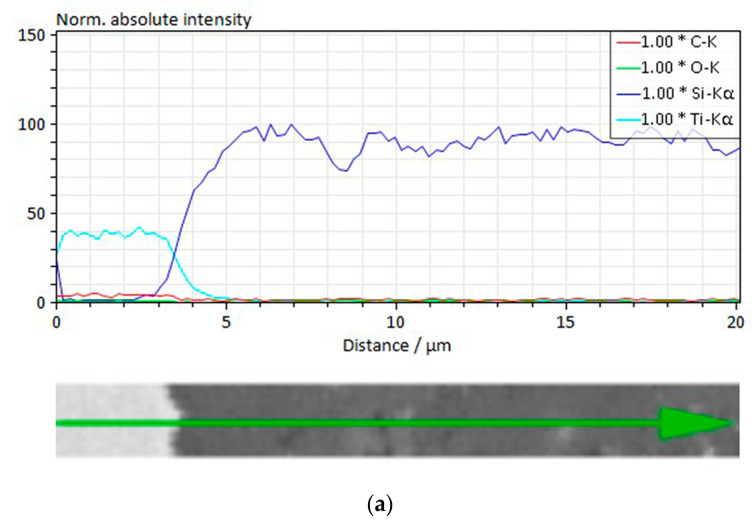

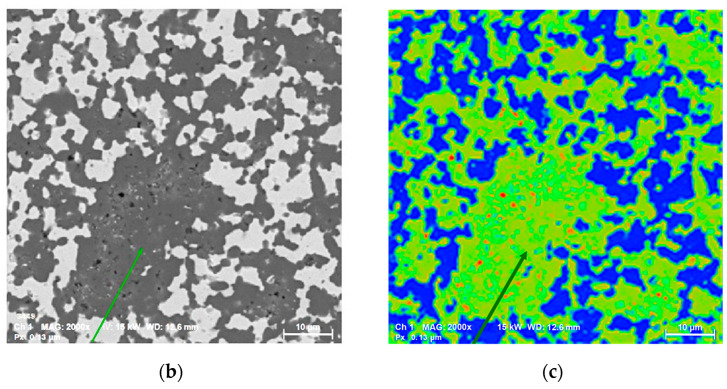
The interaction zone between SiC and TiC phases in the 60 SiC–40 TiC composite: (**a**) M-XRF diagram, (**b**) the analyzed area with the sampling direction marked with a green arrow, and (**c**) a heat map facilitating the more precise delineation of the sample granularity and boundaries. The color scale ranges from red to blue with thresholds set at every 10% increment in image brightness.

A micro-X-ray fluorescent spectroscopy (M-XRF) analysis of the grain boundaries between phases revealed that under a uniaxial pressure of 45 MPa, the interaction zone covered ca. 1.5 µm. Figure 4a shows the diagram corresponding to the radiation registered along the green arrow shown in Figure 4b. This feature can be attributed to a certain non-stoichiometry of TiC [36] and the appearance of very small amounts of a liquid phase in the Si–Ti–C system [37,38].

**Figure 5:**
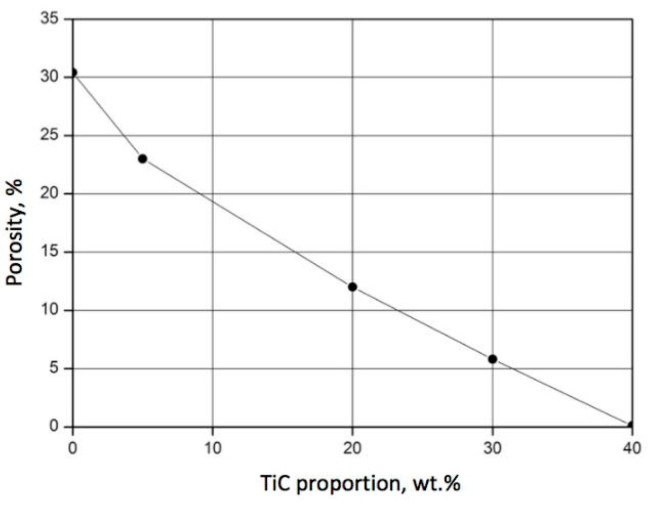
Effect of TiC content on porosity of material sintered at 2000 °C under uniaxial pressure of 45 MPa for 30 min.

The proportion of titanium carbide directly affected the porosity of the SiC–TiC composites. It was found that an increase in TiC content in the powder mixture resulted in a decreased porosity of the sintered material when all other parameters of the process were kept unchanged. The diagram shown in Figure 5 reveals the dependence of porosity on the TiC content for composites sintered at 2000 °C under a uniaxial pressure of 45 MPa for 30 min. It is noteworthy that when the proportion of titanium carbide reached 40 wt.%, the porosity was reduced to almost 0%.

**Figure 6:**
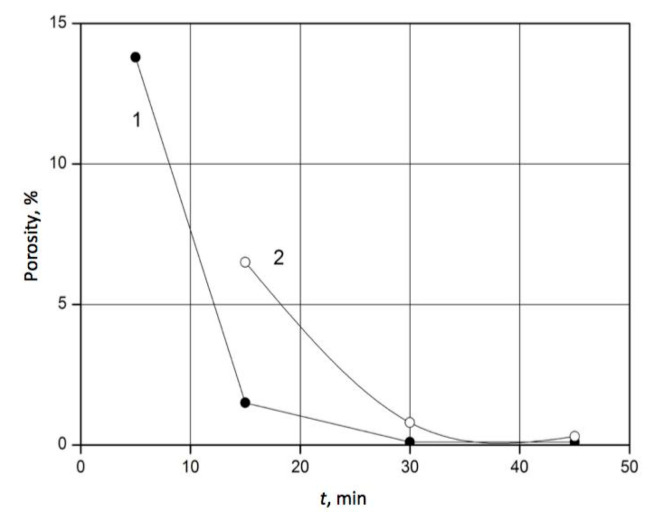
Effect of the holding time on the porosity of the 60 wt.% SiC–40 wt.% TiC material sintered under a uniaxial pressure of 45 MPa at the following temperatures: 1–T = 2000 °C (●); 2–T = 1900 °C (○).

Conversely, the porosity of the sintered material is directly dependent on the holding time t and sintering temperature T. The corresponding diagrams for the composition of 60 wt.% SiC–40 wt.% TiC are presented in Figure 6. After 30 min, porosity was reduced below 1%, and for longer holding times, the sintering temperature made no difference.

### 3.2. Mechanical Properties of the Composite

**Figure 7:**
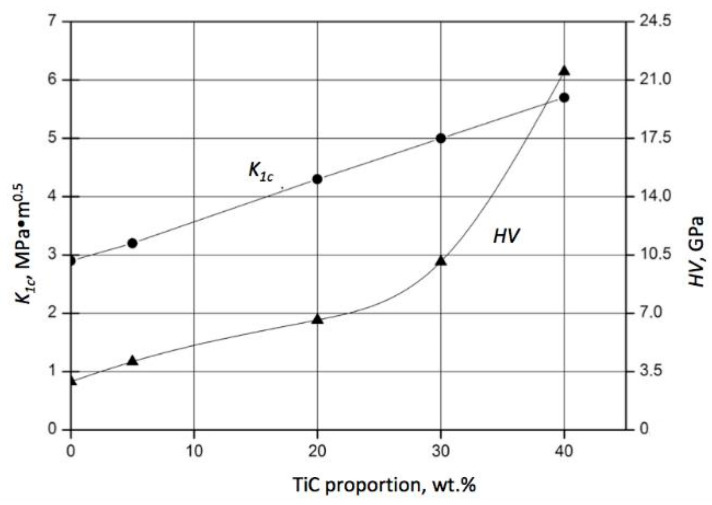
Effect of TiC content on the fracture toughness K_1c_ (marked ●) and hardness HV (marked ▲) of SiC–TiC composites sintered at 2000 °C under a uniaxial pressure of 45 MPa for 30 min. (The standard deviation of K_1c_ and HV varied from 0.3 to 0.5 MPa·m^0.5^ and from 0.8 to 1.0 GPa, respectively).

The complex interaction between SiC and TiC influenced not only the porosity but also the mechanical properties of the composite. An increase in both fracture toughness K_1c_ and hardness HV was registered for a higher proportion of TiC, as shown in Figure 7.

The improvement in fracture toughness with increased TiC addition can be attributed to the mismatch between thermal expansion coefficients α–SiC = 4.8  × 10^−6^/°K and α–TiC = 7.4  × 10^−6^/°K and to the related residual stresses [39]. It was found that the addition of TiC in a proportion of 20 wt.% to the SiC powder of a 2 µm particle size resulted in an increase in the fracture toughness of the sintered composite from 2.9 MPa⋅m^0.5^ up to 4.3 MPa⋅m^0.5^, while hardness HV increased from 2.9 GPa up to 6.6 GPa. A further increase in TiC content caused an almost proportional enhancement in fracture toughness, which reached K_1c_ = 5.7^.^ MPa⋅m^0.5^ for 40 wt.% of TiC. On the other hand, a twofold increase in the TiC content from 20 wt.% up to 40 vol.% resulted in a threefold increase in hardness HV up to 21.5 GPa. When the composite was sintered at lower temperatures (1900 °C), the respective values of toughness and hardness were reduced by ca. 25%. However, a further increase in the sintering temperature caused the significant worsening of the mechanical properties of the composites. This phenomenon can be explained by extensive SiC grain growth at higher temperatures.

**Figure 8:**
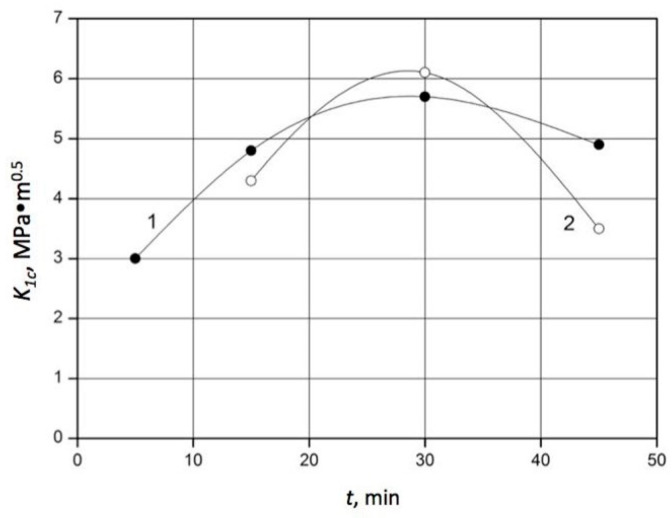
Effect of the holding time on the fracture toughness K_1c_ of the 60 wt.% SiC–40 wt.% TiC material sintered at the following temperatures: 1–T = 2000 °C (●); 2–T = 1900 °C (○). (The standard deviation of K_1c_ varied from 0.3 to 0.5 MPa·m^0.5^).

**Figure 9:**
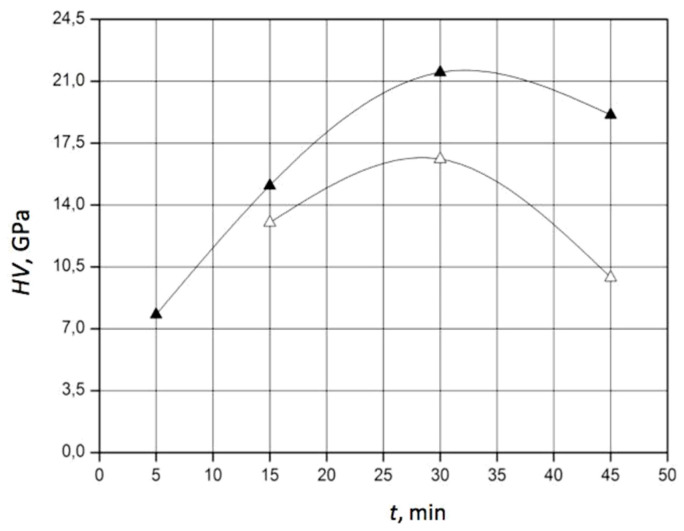
Effect of the holding time on the hardness HV of the 60 wt.% SiC–40 wt.% TiC material sintered under a uniaxial pressure of 45 MPa at the following temperatures: 1–T = 2000 °C (▲); 2–T = 1900 °C (△). (The standard deviation of HV varied from 0.8 to 1.0 GPa).

It should be noted that an improvement in the fracture toughness of a composite is accompanied by an increase in hardness. The composite 60 wt.% SiC–40 wt.% TiC–40 TiC exhibited better properties compared to the results for pressureless sintering reported in [40], where the highest hardness of 20 GPa was found in samples containing 5 vol.% TiC, and a fracture toughness of 5.5 MPa⋅m^0.5^ was reached in samples containing 10 vol.% TiC, sintered at 1885 °C for 1 h. Despite the larger grain sizes compared to nanoscale initial powders, as well as the lower temperatures used in [41], the fracture toughness reached in this study for the 60 wt.% SiC–40 wt.% TiC material was 15% higher than that of SiC sintered under 25 GPa at 1500 °C. Simultaneously, a hardness above 21 GPa was reached, typical for titanium carbide matrix composites [42]. Further analysis revealed a dependence of the composite’s toughness and hardness on the holding time at different temperatures. In particular, the diagrams of K_1c_ and HV for the composition 60 wt.% SiC–40 wt.% TiC sintered at 1900 °C and 2000 °C are presented in Figure 8 and Figure 9, respectively.

The experimental results show that after the sintering of the composite 60 wt.% SiC–40 wt.% TiC at a temperature of 1900 °C for 15 min, fracture toughness K_1c_ increased up to 3.5 MPa⋅m^0.5^, while hardness HV increased up to 9.9 GPa. When the holding time was prolonged up to 30 min at a sintering temperature of 1900 °C, K_1c_ increased up to 6.1 MPa⋅m^0.5^, while HV increased up to 16.6 GPa. The further prolongation of the holding time up to 45 min caused a decrease in fracture toughness down to 3.5 MPa⋅m^0.5^ and a decrease in hardness down to 9.9 GPa. Apparently, this was a consequence of the increased sizes of silicon carbide grains. The sintering of the composite 60 wt.% SiC–40 wt.% TiC at a temperature of 2000 °C after 15 min provided a fracture toughness of 4.8 MPa⋅m^0.5^ and a hardness of 15.1 GPa. An increase in the holding time up to 30 min caused significant enhancements in crack resistance to 5.7 MPa⋅m^0.5^ and in hardness to 21.5 GPa. However, after the sintering of this composite for 45 min, a substantial drop in the toughness and hardness of the composite was observed, caused by the growth of silicon carbide grains.

The literature review indicated that systematic studies of both HV and K_1c_ in relation to changes in TiC percentage in SiC–TiC composites had only been conducted by Ueki et al. [43]. In the aforementioned study, the researchers used a different technique—hot pressing in an Ar inert gas atmosphere at a temperature of 2150 °C and a pressure of 40 MPa for 2 h. The starting material was β–SiC powder, which was transformed in this process to a high-temperature phase. The authors obtained a composite that exhibited higher HV but lower K_1c_. Furthermore, the composite displayed a higher level of porosity in comparison with the material produced in this research. This outcome is most likely attributable to elevated temperatures exceeding 2000 °C, a condition that has been demonstrated to be deleterious. In the time-consuming process proposed by Ueki et al. [43], the optimum doping level was ascertained to be 50%. However, the lower doping level proposed in this study is more advantageous because higher TiC admixture results in low strength (internal brittleness). It is evident that the properties of composites obtained by Chang et al. [44] and Cabrero et al. [45] are also inferior.

### 3.3. Material Feature Vectors Describing the Mechanical Properties of the Composite

As follows from the analysis of the experimental data, the combination of porosity, fracture toughness, and hardness is responsible for the unique and highly desirable properties of the composites. However, the process of comparing individual material features to identify or predict the optimal set of features may be time-consuming. To improve its efficiency, the use of a material feature vector, characterizing the structure and mechanical properties of composites, was proposed. The process of evaluating and quantifying the similarity between material feature vectors describing different SiC–TiC composites constitutes a pivotal element in both the formulation of recommendations and the identification of anomalies.

Each value in the 2D and 3D material feature vectors corresponds to a specific property of the sample under analysis: K_1c_ and HV or porosity, K_1c_, and HV, respectively.

The magnitude of the material feature vector and the angle between vectors, presented in Table 3, increased with the increase in TiC content. The sole exception is the TiC proportion 30 wt.%, which serves to demonstrate that both length and angle must be considered.

**Figure 10:**
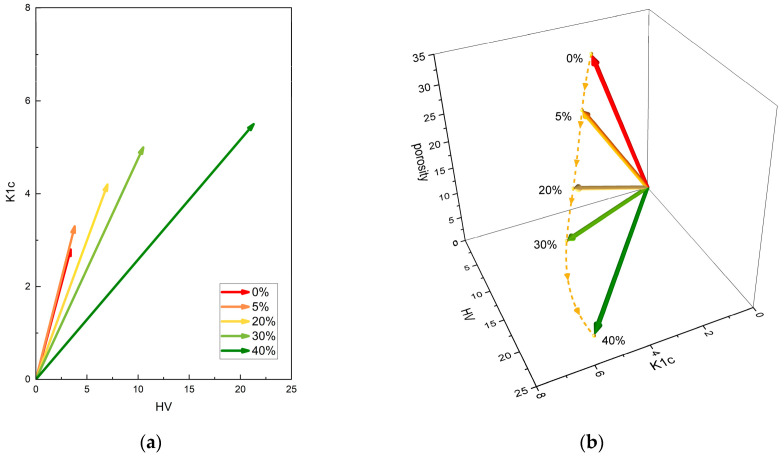
A visualization of the feature vector in the material feature space: (**a**) 2D and (**b**) 3D.

The vector spaces associated with these feature vectors, so-called 2D and 3D material feature spaces, are shown in Figure 10a and Figure 10b, respectively.

The path that follows the vector at any given point (trajectory), as shown in Figure 10b, describes the change in the physical properties of the material upon a change in TiC content in the composite. Based on this trajectory, the physical values at intermediate points, i.e., for other percentages of TiC, can be easily interpolated. The higher the angle, the better the material—i.e., the more the vector deviates from the K_1c_/HV plane, the worse the properties the composite exhibits will be.

**Figure 11:**
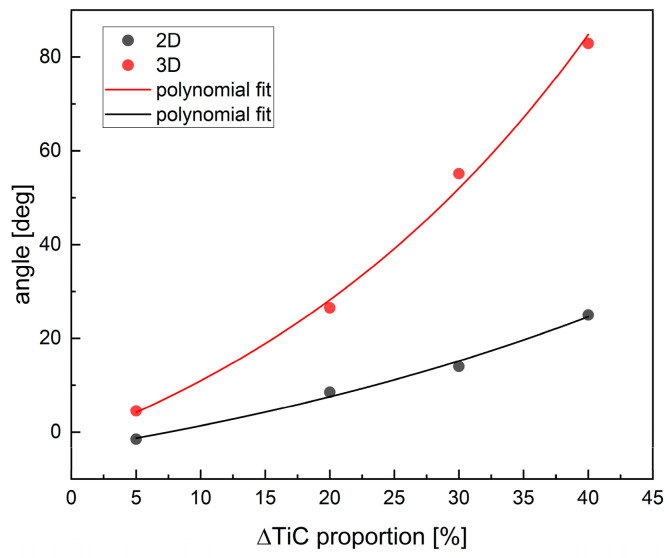
The angle between the feature vector describing pure TiC and the 5, 20, 30, and 40% percentage admixtures of TiC, respectively.

The variation in the feature vector orientation with increasing TiC content is shown in Figure 11.

The alteration in the orientation of the feature vector in space upon an increase in TiC content is characterized by a monotonic progression. Porosity exerts a considerably more pronounced effect on the angle changes.

**Figure 12:**
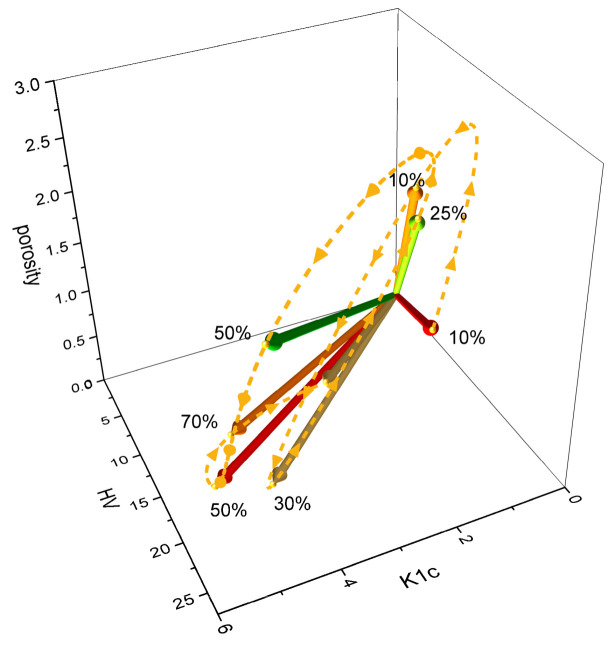
A visualization of the material feature vector in the 3D material feature space, with data from Refs. [43,45].

The changes in the orientation of the material feature vector in space upon an increase in TiC percentage (visualized based on data from [43,45]) are non-monotonic (Figure 12).

The trajectories (marked in yellow) illustrate the differences in the physical properties of the material resulting from the variation in the TiC content within the composite, as observed in both studies. They demonstrate the challenges associated with obtaining composites that exhibit a continuous transition in properties upon TiC percentage increase. However, the more deviated the vector was from the K_1c_/HV plane, the worse the obtained composite properties were. This simple principle remains applicable.

In the context of material recommendation, two feature vectors describing preferences should be similar if they share common features or lead to the same choices. Comparisons of their similarity estimated using metrics that consider magnitude and direction (Euclidean distance, ED) or direction (cosine similarity, CS) can be helpful. As the ED value increases, the discrepancy between vectors becomes more pronounced. Conversely, as the CS decreases, the similarity between vectors becomes more apparent. A value of CS closer to 1 indicates a greater level of similarity, while a value closer to 0 indicates no similarity. (The margin of error pertaining to distances does not exceed 5%.)

The ED and CS distances between each pair of the 2D and 3D feature vectors are summarized in a tabular form and colored using a heat mapping technique in Table 4 and Table 5.

**Figure 13:**
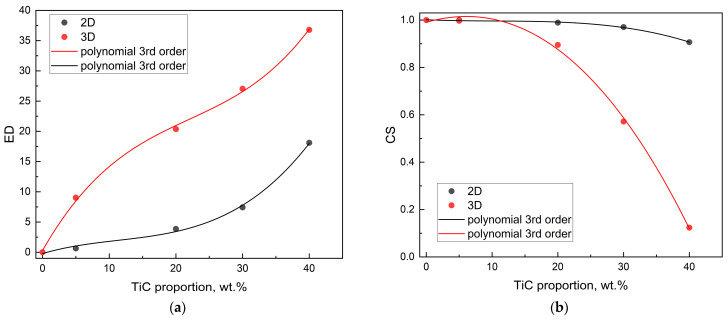
The similarity of the material feature vectors versus TiC proportion: (**a**) Euclidean distance; (**b**) cosine similarity.

Optimization requires a different approach from material feature vector variability, as the proximity of vectors is no longer a desirable feature. Conversely, it has been demonstrated that an increase in distance is directly correlated with an enhancement in material properties. As TiC content increases, the discrepancy between the ED similarity values becomes more pronounced (Figure 13a), but the CS similarity becomes more apparent (Figure 13b).

As follows from the above data, the 3D material feature vector is much more sensitive to the TiC proportion than the 2D material feature vector. Thus, the effect of porosity is significant and well reproduced by the 3D feature vector. The changes in the orientation of the feature vector in space and the similarity measures of material feature vectors indicate changes of a monotonic nature caused by the increase in the percentage of TiC.

A range of techniques exists for the manufacturing of composite materials, which in turn leads to significant fluctuations in material features. Consequently, it is challenging to predict the optimal percentage of admixtures for the manufacturing of composite materials. (The repeatability of materials obtained using such techniques is often poor.) In contrast to other methods, the behavior of the 2D and 3D material feature vectors for the SiC–TiC composites obtained by electroconsolidation is monotonic. This finding indicates that the material properties of SiC–TiC exhibit a smooth transition in response to TiC percentage.

Similar analysis was performed based on the data collected with different holding times and temperatures (Table 6).

**Figure 14:**
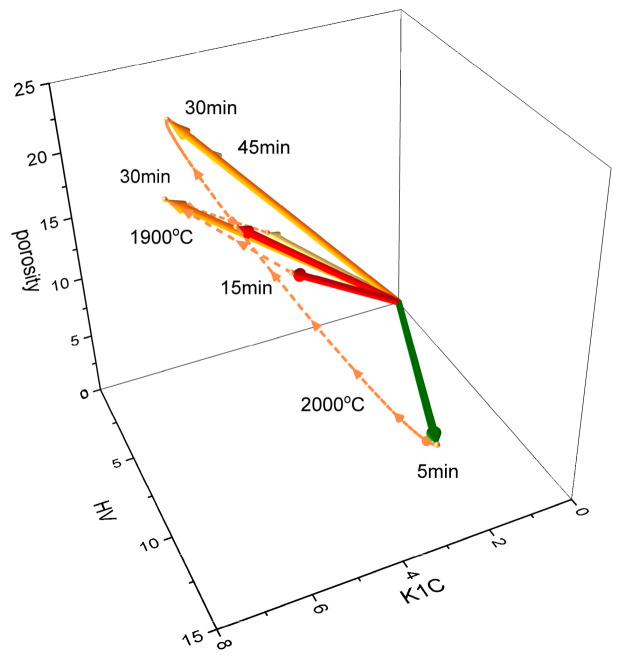
A visualization of the influence of holding time on the 3D material feature vectors.

A visualization of the material feature vectors in 3D material feature space is shown in Figure 14.

The highest magnitude of 2D and 3D material feature vectors indicates the best option. The clustered nature of vectors at 1900 °C and their scattered nature at 2000 °C illustrate the role that holding time plays in composite features.

The Euclidean distance and cosine similarity between material feature vectors at different holding times (T = 2000 °C and 1900 °C) are summarized in tabular form and colored using a heat mapping technique in Table 7, Table 8, Table 9 and Table 10.

The highest ED (Table 7 and Table 9) indicates the optimal holding time. Thus, the effect of holding time is significant and well described by both the 2D and 3D feature vectors. CS distance is not as good a measure of holding time changes as ED, which can be seen in Table 8 and Table 10. The smallest CS should indicate the best option, but the 3D CS points to 45 min instead of 30 min, which is a result of the magnitude of the vectors being neglected by cosine metrics. Consequently, the CS is of limited use in this context.

A comparison of the ED at two different temperatures (Table 6 and Table 8) facilitates the determination of the optimal temperature, that is, 2000 °C. The disparity between the ED values of 10.44 and 7.69, as well as 13.72 and 10.94, for the two-dimensional (2D) and three-dimensional (3D) vector spaces, respectively, is substantial.

## 4. Conclusions

The objective of the present research was to study the influence of the titanium carbide proportion in the SiC–TiC system, sintered under a uniaxial pressure of 45 MPa, on its mechanical properties. Additionally, the investigation delved into the optimization of the aforementioned process, with a view to enhancing its efficiency and efficacy.

It was found that the addition of TiC to silicon carbide reduced porosity, almost reaching full density when the titanium carbide proportion was 40 wt.%. Simultaneously, an increase in the TiC content caused an enhancement in fracture toughness from 2.9 MPa⋅m^0.5^ (for SiC with no TiC addition) to 6.1 MPa⋅m^0.5^ (for 60 wt.% SiC–40 wt.% TiC composite). The respective values of hardness increased significantly from 2.9 GPa up to 21.5 GPa.

Regarding the temperature effect, an increase in the sintering temperature from 1900 °C to 2000 °C caused an increase in fracture toughness and hardness by approximately 30%. Nevertheless, elevated temperatures of 2100 °C resulted in deleterious effects, manifesting as diminished grain growth and the subsequent deterioration of mechanical properties. A similar effect was observed when the holding time was extended up to 45 min. Following a period of 30 min, it was established that full density had almost been attained and that the characteristics of the sintered composites had reached their maximum levels.

The use of two- and three-dimensional vector spaces of material features was proposed as a novel methodology for the description of manufacturing process optimization. The analysis of the 2D and 3D individual material feature vectors and Euclidean distances between them confirmed the choice of the optimal TiC content, holding time, and temperature achieved through a manual comparison of features. (The cosine similarity disregards the magnitude of the vectors and is thus of limited use.) This new approach facilitates more rapid process optimization. The high heating rates, short holding times, and relatively low sintering temperatures required by electroconsolidation ensure a significant reduction in resource usage. It was demonstrated that the process of densification is significantly accelerated using electroconsolidation.

Furthermore, the changes in both the orientation and magnitude of 2D and 3D material feature vectors for SiC–TiC composites with different TiC percentages are monotonic. This finding indicates that the properties of SiC–TiC composites obtained by electroconsolidation show a smooth change in response to the modification of the percentage of TiC content, which distinguishes this method from classical ones such as SPS or HP. Thus, the material feature vector approach revealed the smooth characteristics offered by the manufacturing process, an advantage that is not offered by the classical methods of SPS or HP. Therefore, it can be deduced that electroconsolidation is associated with more predictable outcomes. It can be hypothesized that material feature vector space may offer enhanced process control. However, this remains to be investigated.

## Figures and Tables

**Table 1:** The composition of SiC.

	Spectrum 1	Spectrum 2	Spectrum 3	Average	Standard Deviation
C	59.94	59.30	54.99	58.08	2.69
O	4.17	4.58	4.17	5.03	1.15
Si	35.89	36.12	35.89	36.90	1.54

**Table 2:** The composition of the TiC additive.

	Spectrum 11	Spectrum 10	Spectrum 9	Spectrum 8	Average	Standard Deviation
C	30.67	23.07	26.28	23.42	25.86	3.51
O	—	15.02	—	—	—	—
Al	0.36	0.38	0.33	0.27	0.34	0.05
Ti	68.96	56.64	73.39	76.30	68.82	8.66
Fe	—	4.88	—	—	—	—

**Table 3:** The 2D and 3D material feature vectors and angular distance (in degrees) between TiC 0% and four different TiC contents, namely, 5, 20, 30, and 40%. (The margin of error is no greater than 5%.)

TiC Content [%]	2D $\left[K_{1c},\;HV\right]$		3D $\left[Porosity,\;K_{1c},\;HV\right]$	
	Magnitude	Angle	Magnitude	Angle
0	4.40	—	32.30	—
5	5.03	−1.50	23.54	4.51
20	8.16	8.51	14.51	26.50
30	11.63	14.01	13.09	55.12
40	22.00	24.99	22.00	82.90

**Table 4:** Euclidean distance between material feature vectors: 0, 5, 20, 30, and 40% percentage admixtures of TiC. A red–yellow–green scheme, where green indicates a maximum value and red indicates a minimum value, is used.

2D ED	0	5	20	30	40	3D ED	0	5	20	30	40
0	0	0.64	3.86	7.43	18.1	0	0	9.02	20.37	27.04	36.77
5	0.64	0	3.32	6.91	17.64	5	9.02	0	11.49	18.35	28.98
20	3.86	3.32	0	3.59	14.36	20	20.37	11.49	0	6.99	18.71
30	7.43	6.91	3.59	0	10.81	30	27.04	18.35	6.99	0	13.09
40	18.1	17.64	14.36	10.81	0	40	36.77	28.98	18.71	13.09	0

**Table 5:** Cosine similarity between material feature vectors: 0, 5, 20, 30, and 40% percentage admixtures of TiC. A red–yellow–green scheme, where green indicates a maximum value and red indicates a minimum value, is used.

2D CS	0	5	20	30	40	3D CS	0	5	20	30	40
0	1	0.9997	0.989	0.9703	0.9064	0	1	0.9969	0.8949	0.5718	0.1236
5	0.9997	1	0.9848	0.9636	0.895	5	0.9969	1	0.9261	0.631	0.1913
20	0.989	0.9848	1	0.9954	0.9589	20	0.8949	0.9261	1	0.8767	0.5393
30	0.9703	0.9636	0.9954	1	0.9817	30	0.5718	0.631	0.8767	1	0.8724
40	0.9064	0.895	0.9589	0.9817	1	40	0.1236	0.1913	0.5393	0.8724	1

**Table 6:** The influence of the holding time on the 2D and 3D material feature vectors and their angular distances (in degrees). (The margin of error is no greater than 5%.)

**Holding Time, Min**	2000 °C				1900 °C			
	2D $\left[K_{1c},\;HV\right]$		3D $\left[Porosity,\;K_{1c},\;HV\right]$		2D $\left[K_{1c},\;HV\right]$		3D $\left[Porosity,\;K_{1c},\;HV\right]$	
	Magnitude	Angle	Magnitude	Angle	Magnitude	Angle	Magnitude	Angle
5	7.80	—	16.03	—	—	—	—	—
15	15.08	4.06	15.38	49.72	13.66	—	14.92	—
30	21.86	7.50	21.86	59.84	17.31	2.73	17.38	18.94
45	18.66	7.39	18.66	61.14	10.41	1.75	10.41	23.77

**Table 7:** Euclidean distance between material feature vectors at different holding times (cells are colored to denote varying degrees of magnitude; T = 2000 °C). A red–yellow–green scheme, where green indicates a maximum value and red indicates a minimum value, is used.

2D ED	5	15	30	45	3D ED	5	15	30	45
5	0	7.32	14.16	10.97	5	0	13.22	19.56	17.78
15	7.32	0	6.86	3.70	15	13.22	0	7.30	4.76
30	14.16	6.86	0	3.20	30	19.56	7.30	0	3.24
45	10.97	3.70	3.20	0	45	17.78	4.76	3.24	0

**Table 8:** Cosine similarity between material feature vectors at different holding times (cells are colored to denote varying degrees of magnitude; T = 2000 °C). A red–yellow–green scheme, where green indicates a maximum value and red indicates a minimum value, is used.

2D CS	5	15	30	45	3D CS	5	15	30	45
5	1	0.9975	0.9914	0.9917	5	1	0.6466	0.5024	0.4827
15	0.9975	1	0.9982	0.9983	15	0.6466	1	0.9832	0.9791
30	0.9914	0.9982	1	1.0000	30	0.5024	0.9832	1	0.9997
45	0.9917	0.9983	1.0000	1	45	0.4827	0.9791	0.9997	1

**Table 9:** Euclidean distance between material feature vectors at different holding times and 1900 °C. A red–yellow–green scheme, where green indicates a maximum value and red indicates a minimum value, is used.

2D ED	15	30	45	3D CS	15	30	45
15	0	3.72	3.28	15	0	5.84	6.84
30	3.72	0	6.91	30	5.84	0	7.07
45	3.28	6.91	0	45	6.84	7.07	0

**Table 10:** Cosine similarity between material feature vectors at different holding times and 1900 °C. A red–yellow–green scheme, where green indicates a maximum value and red indicates a minimum value, is used.

2D CS	15	30	45	3D CS	15	30	45
15	1.0000	0.9989	0.9995	15	1.0000	0.9459	0.9152
30	0.9989	1.0000	0.9999	30	0.9459	1.0000	0.9961
45	0.9995	0.9999	1.0000	45	0.9152	0.9961	1.0000

## Data Availability

All data are incorporated into the manuscript.

## References

[1] Zhu B.P., Go W.K., Shen G.Z., Zhou Q., Sung K.K. (2011). Structure and electrical properties of (111)-oriented Pb(Mg_1/3_Nb_2/3_)O_3_-PbZrO_3_-PbTiO_3_ thin film for ultra-high-frequency transducer applications. IEEE Trans. Ultrason. Ferroelectr. Freq. Control.

[2] Zhang W., Shima H., Takano F., Takenaka M., Akinaga H., Nimori S. (2009). Magnetron sputtering deposition of Pr_12.5_Fe_77.5_B_10_ thin films and substrate temperature dependence of the magnetic properties. J. Phys. D.

[3] Zhu B.P., Fei C., Wang C., Zhu Y., Yang X., Zheng H., Zhou Q., Shung K.K. (2017). Self-Focused AlScN Film Ultrasound Transducer for Individual Cell Manipulation. ACS Sens..

[4] Zhu B.P., Zhang Z., Ma T., Yang X., Li Y., Shung K.K., Zhou Q. (2015). (100)-Textured KNN-based thick film with enhanced piezoelectric property for intravascular ultrasound imaging. Appl. Phys. Lett..

[5] Zhu B.P., Wang Z., Zhang Y., Yu Z.S., Shi J., Xiong R. (2009). Low temperature fabrication of the giant dielectric material CaCu_3_Ti_4_O_12_ by oxalate coprecipitation method. Mater. Chem. Phys..

[6] Sui X., He W., Zuo C., Chen Q., Gu G. (2014). 10.6 μm Infrared light photoinduced insulator-to-metal transition in vanadium dioxide. Infrared Phys. Technol..

[7] Choyke W.J., Matsunami H., Pensl G. (2004). Silicon Carbide: Recent Major Advances.

[8] La Via F., Alquier D., Giannazzo F., Kimoto T., Neudeck P., Ou H., Roncaglia A., Saddow S.E., Tudisco S. (2023). Emerging SiC Applications beyond Power Electronic Devices. Micromachines.

[9] Nava F., Vanni P., Bruzzi M., Lagomarsino S., Sciortino S., Wagner G., Lanzieri C. (2004). Minimum ionizing and alpha particles detectors based on epitaxial semiconductor silicon carbide. IEEE Trans. Nucl. Sci..

[10] Bernat R., Capan I., Bakrač L., Brodar T., Makino T., Ohshima T., Pastuović Ž., Sarbutt A. (2021). Response of 4H-SiC Detectors to Ionizing Particles. Crystals.

[11] Lei X., Kane S., Cogan S., Lorach H., Galambos L., Huie P., Mathieson K., Kamins T., Harris J., Palanker D. (2016). SiC protective coating for photovoltaic retinal prosthesis. J. Neural Eng..

[12] Zorman C.A., Parro R.J. (2008). Micro- and nanomechanical structures for silicon carbide MEMS and NEMS. Phys. Stat. Sol..

[13] Lohrmann A., Iwamoto N., Bodrog Z., Castelletto S., Ohshima T., Karle T.J., Gali A., Prawer S., McCallum J.C., Johnson B.C. (2015). Single-photon emitting diode in silicon carbide. Nat. Commun..

[14] Castelletto S. (2021). Silicon carbide single-photon sources: Challenges and prospects. Mater. Quantum Technol..

[15] Aldalbahi A., Li E., Rivera M., Velazquez R., Altalhi T., Peng X., Feng P.X. (2016). A new approach for fabrications of SiC based photodetectors. Sci. Rep..

[16] Cheung R. (2006). Silicon Carbide Microelectromechanical Systems for Harsh Environments.

[17] Heuer A.H., Fryburg G.A., Ogbuji L.U., Mitchell T.E., Shinozaki S. (1978). Beta-alpha transformation in polycrystalline SiC: 1, microstructural aspects. J. Am. Ceram. Soc..

[18] Dresch A.B., Venturini J., Arcaro S., Montedo O.R.K., Bergmann C.P. (2021). Ballistic ceramics and analysis of their mechanical properties for armour applications: A review. Ceram. Int..

[19] Chen B., Niu L., Chai J., Bai Z., Zhang J., Liu D., Lu X., Zhu Y. (2025). Experimental investigation of microstructure and mechanical properties of β-SiC with various sintering additives supplemented by first-principles calculations. Ceram. Int..

[20] Shin S., Kim M., Kim M., Kim U., Kim S.-G., Kwak Y., Cho J. (2025). Ultrafast high-temperature sintering of reaction-bonded SiC with Y_2_O_3_-Al_2_O_3_ sintering additives. Mater. Lett..

[21] Gevorkyan E.S., Nerubatskyi V.P., Vovk R.V., Chyshkala V.O., Kislitsa M.V. (2022). Structure Formation in Silicon Carbide–Alumina Composites during Electroconsolidation. J. Superhard Mater..

[22] Selvam J.D., Dinaharan I., Rai R.S. (2021). Matrix and Reinforcement Materials for Metal Matrix Composites. Encycl. Mater. Compos..

[23] Jiang D.L., Wang J.H., Li Y.L., Ma L.T. (1989). Studies on the strengthening of silicon carbide-based multiphase ceramics I: The SiC TiC system. Mater. Sci. Eng. A.

[24] Ivzhenko V.V., Gevorkyan E.S., Kosenchuk T.O. (2021). Sintering and Properties of Materials Synthesized on the Basis of Silicon, Boron, and Titanium Carbides by Electrospark Sintering. J. Superhard Mater..

[25] Liu J., Li Y., Cheng C., Li W., Wu W., Jin Y. (2023). Study on the toughening mechanism of in-situ synthesis (Ti_x_Zr_1−x_)B_2_ in solid-state sintered SiC composite ceramics. J. Eur. Ceram. Sci..

[26] Wan C., Xia P., Hua J., Chen X., Lang W., Xue W., Geng Y., Fang T. (2025). Fabrication of SiC composites by selective laser sintering and reactive melt infiltration. Ceram. Int..

[27] Hao B., Fan L., Li K., Fu Y., Guo X., Guan L., Yang S., Zhang M., Sun D., An L. (2024). Hot oscillatory pressing of SiC ceramics with B_4_C and C as sintering additives. Ceram. Int..

[28] Yang C., Li X., Wei Y., Gao Y., Liu M. (2025). Effect of He ions irradiation at 650 °C on microstructural evolution, chemical bonding changes and hardening of pressureless solid-state sintered SiC. Ceram. Int..

[29] Ivzhenko V., Vovk R., Hevorkian E., Kosenchuk T., Chyshkala V., Nerubatskyi V., Cherniavskyi V., Shamsutdinova N. (2025). Investigation of Electrospark Sintering of Composites of SiC–TiC, SiC–VC Systems. Materials.

[30] Liu W., Peng J., Liu J., Li J., Liu B., Fang Q. (2024). Investigation of the sintering behavior of nanoparticulate SiC by molecular dynamics simulation. Mater. Today Commun..

[31] Lei P., Yu M., Gucci F., Huang Z., Fu R., Zhang D. (2024). Numerical simulation of heat transfer during spark plasma sintering of porous SiC. Ceram. Int..

[32] Hevorkian E.S., Nerubatskyi V.P., Rucki M., Kilikevicius A., Mamalis A.G., Samociuk W., Morozow D. (2024). Electroconsolidation Method for Fabrication of Fine-Dispersed High-Density Ceramics. Nanotechnol. Percept..

[33] Piotrkiewicz P., Zygmuntowicz J., Wachowski M., Cymerman K., Kaszuwara W., Midor W.A. (2022). Al_2_O_3_-Cu-Ni Composites Manufactured via Uniaxial Pressing: Microstructure, Magnetic, and Mechanical Properties. Materials.

[34] Latosińska M., Latosińska J.N. (2024). Favipiravir Analogues as Inhibitors of SARS-CoV-2 RNA-Dependent RNA Polymerase, Combined Quantum Chemical Modeling, Quantitative Structure–Property Relationship, and Molecular Docking Study. Molecules.

[35] Latosińska J.N., Latosińska M., Seliger J., Žagar V., Apih T. (2024). Butterfly Effect in Cytarabine: Combined NMR-NQR Experiment, Solid-State Computational Modeling, Quantitative Structure-Property Relationships and Molecular Docking Study. Pharmaceuticals.

[36] Chiu K.A., Lin J.F., Lin K.Y., Wu P.H., Chen H.I., Ko C.J., Chen C.H., Chang L. (2023). Epitaxial growth of TiC on (0001) 4H-SiC substrate by reactive sputtering. Thin Solid Films.

[37] Kamei K., Kusunoki K., Yashiro N., Okada N., Tanaka T., Yauchi A. (2009). Solution growth of single crystalline 6H, 4H-SiC using Si–Ti–C melt. J. Cryst. Growth.

[38] Lapin J., Klimová A., Pelachová T., Štamborská M., Bajana O. (2023). Synergistic effect of Ti, B, Si, and C on microstructure and mechanical properties of as-cast Al_0.4_Co_0.9_Cr_1.2_Fe_0.9_Ni_1.2_(Si, Ti, C, B)_0.375_ complex concentrated alloy. J. Alloys Compd..

[39] Magnus C., Sharp J., Ma L., Rainforth W.M. (2020). Ramification of thermal expansion mismatch and phase transformation in TiC-particulate/SiC-matrix ceramic composite. Ceram. Int..

[40] Ahmoye G., Bucevac D., Krstic V.D. (2018). Mechanical properties of reaction sintered SiC-TiC composite. Ceram. Int..

[41] Sun R., Zhang X., Hao X., Hu W., Wei X., Song X., Zhang Z., Ying P., Zhao S., Wang Y. (2025). Simultaneously enhanced toughness and hardness of nanocrystalline SiC sintered under high pressure. J. Eur. Ceram. Sci..

[42] Lou Z., Li Y., Zou Q., Luo W., Gu H., Li Z., Luo Y. (2023). In-situ fabrication and characterization of TiC matrix composite reinforced by SiC and Ti_3_SiC_2_. Ceram. Int..

[43] Ueki M., Endo H. (1992). Mechanical Properties of Particulate Reinforced SiC-based Ceramic Composites. ISIJ Int..

[44] Cheng L.X., Xie Z.P., Liu G.W. (2013). Spark plasma sintering of TiC-based composites toughened by submicron SiC particles. Ceram. Int..

[45] Cabrero J., Audubert F., Pailler R. (2011). Fabrication and characterization of sintered TiC-SiC composites. Eur. Ceram. Soc..

